# Consequences of the closure of general practices: a retrospective cross-sectional study

**DOI:** 10.3399/BJGP.2022.0501

**Published:** 2023-05-16

**Authors:** Joseph Hutchinson, Kath Checkland, Jon Gibson, Evangelos Kontopantelis, Matt Sutton

**Affiliations:** Centre for Primary Care and Health Services Research, University of Manchester, Manchester.; Centre for Primary Care and Health Services Research, University of Manchester, Manchester.; Centre for Primary Care and Health Services Research, University of Manchester, Manchester.; Centre for Primary Care and Health Services Research, University of Manchester, Manchester.; Centre for Primary Care and Health Services Research, University of Manchester, Manchester.

**Keywords:** general practice, health services research, primary health care, quality of health care, workforce

## Abstract

**Background:**

Two general practices close every week in the UK. Given the pressure on UK general practices, such closures are likely to persist. Yet little is known about the consequences. Closure refers to when a practice ceases to exist, merges, or is taken over.

**Aim:**

To explore whether practice funding, list size, workforce composition, and quality change in surviving practices when surrounding general practices close.

**Design and setting:**

A cross-sectional study of English general practices was undertaken, using data from 2016–2020.

**Method:**

The exposure to closure for all practices existing on 31 March 2020 was estimated. This is the estimation proportion of a practice’s patient list that had been through a closure in the preceding 3 years, between 1 April 2016 and 3 March 2019. The interaction between the exposure to closure estimate and the outcome variables (list size, funding, workforce, and quality) was analysed through multiple linear regression, while controlling for confounders (age profile, deprivation, ethnic group, and rurality).

**Results:**

A total of 694 (8.41%) practices closed. A 10% increase in exposure to closure resulted in 1925.6 (95% confidence interval [CI] = 1675.8 to 2175.4) more patients in the practice with £2.37 (95% CI = £4.22 to £0.51) less funding per patient. While numbers of all staff types increased, there were 86.9 (95% CI = 50.5 to 123.3), 4.3%, more patients per GP. Increases for other staff types were proportionate to increases in patients. Patient satisfaction with services declined across all domains. No significant difference in Quality and Outcomes Framework (QOF) scores was identified.

**Conclusion:**

Higher exposure to closure led to larger practice sizes in remaining practices. Closure of practices changes workforce composition and reduces patient satisfaction with services.

## INTRODUCTION

Two general practices are closing every week in the UK.^1^ General practices are the principal providers of primary care within the UK, meaning that closures have impacted millions of patients.^1^

Closure principally refers to organisations ceasing to exist or changing ownership.^2^ A change of ownership is when two or more companies combine, either via negotiation, a merger, or by acquisition, a takeover.^3^ Closure can also refer to financial failure, comprising bankruptcy, restructuring, and financial underperformance.^2^ However, more than 90% of hospitals in financial distress do not cease to exist or change ownership,^4^ which makes this a poor definition in health care.

General practice in England consists of partnerships of primary care physicians, known as general practitioners (GPs), who hold a contract with the NHS to provide primary medical services. Every person in the UK is entitled to register with a single practice, bringing funding of £99.70 per patient. This funding is weighted for variations in workload dependent on patient characteristics such as age.^5^ Practices then act as the gatekeepers to the wider NHS. Beyond this core funding, practices can also opt into other funding streams. This includes a pay-for-performance scheme, known as the Quality and Outcomes Framework (QOF), 6 as well as the provision of additional services, known as enhanced services.^7^

Closures of UK general practices accelerated from less than one a week in 2013 to around six a week in 2018, before declining to around four a week in 2020. This is alongside a gradual increase in average list size from 6914 to 9007 between 2013 and 2020, with 1398 fewer practices overall.^8^ This suggests a gradual consolidation of patients into expanding practices over the previous decade, which is in concordance with the general practice policy environment, where practices are encouraged to serve larger populations such as primary care networks.^9^^,^^10^ This potentially brings benefits through scale mechanisms and resilience from GP retirements. However, some evidence suggests that these larger practices may have poorer continuity of care, with working at scale not in itself improving patient access or clinical performance.^9^^,^^11^

When ceasing to exist, practices will hand their contract back to the NHS (commissioner), it will then be responsible for offering an alternative practice. In essence, patients will then register at alternative local practices, which may be forced to accept them.^11^ Ownership change is likely to be more complicated, but principally involves the consolidation of multiple contracts into one, with negotiation between the constituent providers.

There is heterogeneity in the existing literature on the consequences of primary care closure, with both increases and decreases in healthcare utilisation, quality, and patient care being identified.^12^^–^18

**Table:** How this fits in

Closures of UK general practices are increasingly common, yet little is known about the consequences. This cross- sectional study of English general practices finds practice closures increase list size in exposed practices, with changes in workforce composition and reductions in patient satisfaction.

Many papers explore physician exits, as opposed to facility closure, which may have different outcomes.^15^^,^^17^ Further, heterogeneity may reflect differences between the health systems studied or closure definitions used. However, despite heterogeneity, this existing literature demonstrates that closures do have impacts on the health system and patient outcomes.

General practice in England is under increasing pressure owing to multiple patient, system, and supply-side factors.^19^ This will likely translate into the persistence or increase in practice closures. However, the consequences of these closures in England need to be clarified. To begin to address this research gap, this study aimed to understand if the closure of English general practices changes the list size, funding, workforce composition, and quality indicators of remaining practices.

## METHOD

### Study design

Utilising a retrospective cross-sectional methodology, practice financial, workforce, quality, demographic, and neighbourhood data were linked at practice level for English general practices for the 2019–2020 financial year. The 2019–2020 financial year was chosen to avoid any influence from the COVID-19 pandemic.

### Closure exposure coefficient

**Figure 1.**
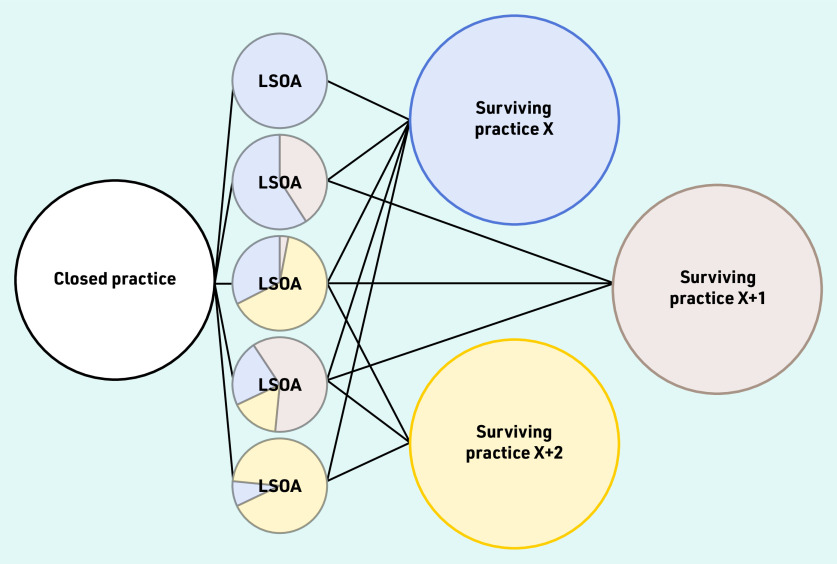
*Example of patient flow following a practice ceasing to exist using LSOA patient grouping.LSOA = Lower layer Super Output Areas.*

**Figure 2.**
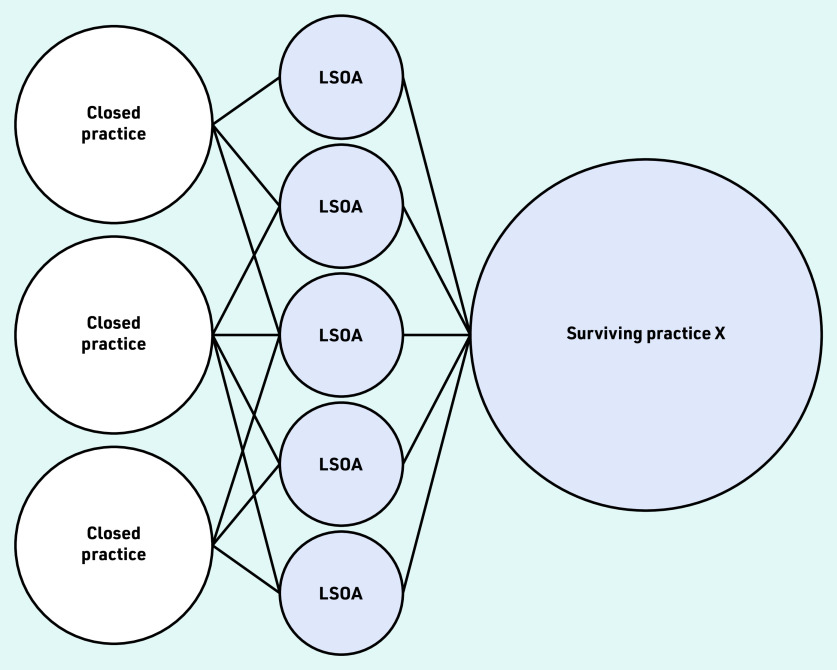
*Example of patient flow following a practice merging or being taken-over using LSOA patient grouping. LSOA = Lower layer Super Output Areas.*

**Figure 3.**
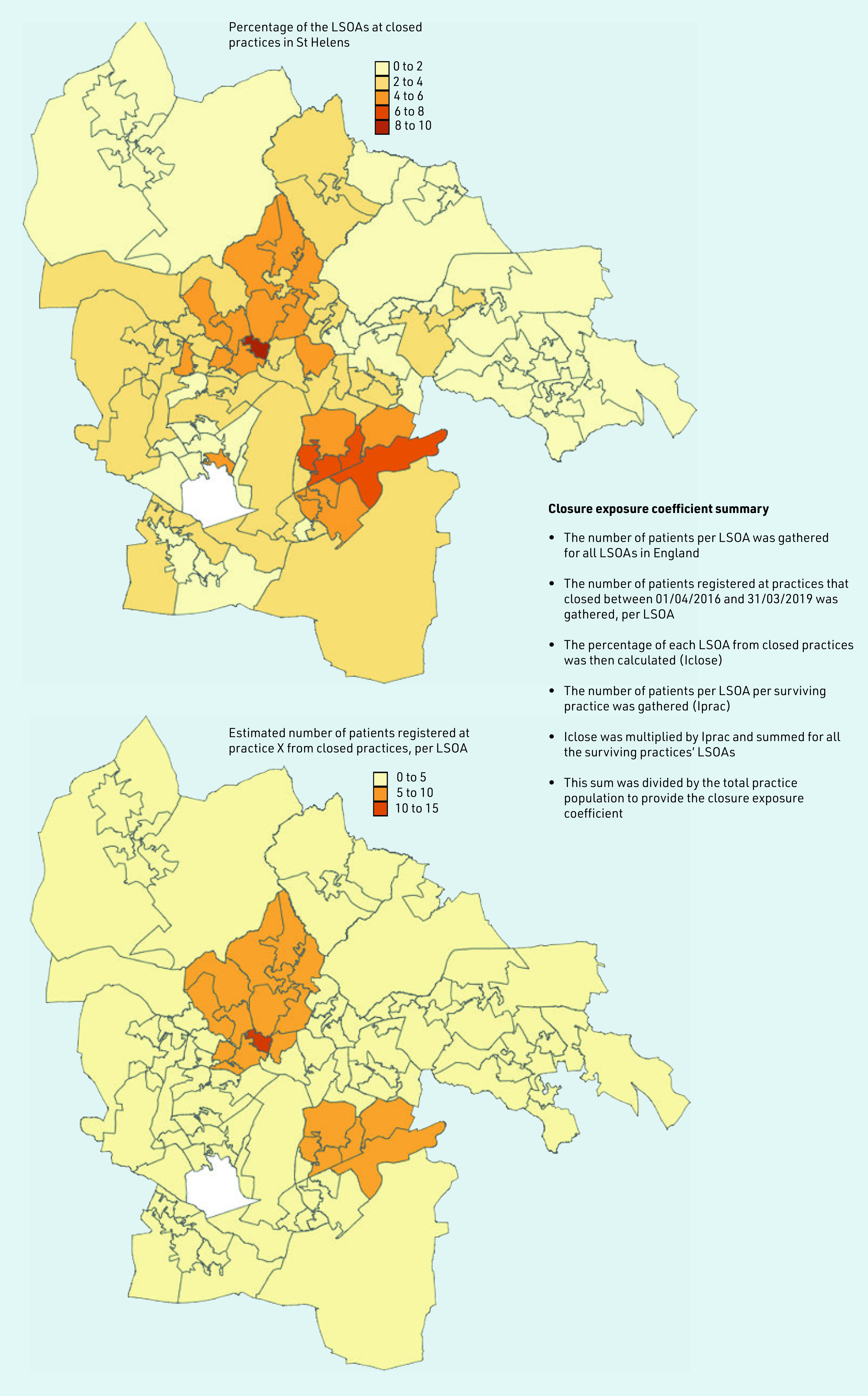
*Closure exposure coefficient equation and diagrams for an example practice X in St Helens.*

The closure of a practice was gathered from NHS Digital’s Organisation Data Service. It maintains a complete list of practices in England and Wales. It codes practices as either open, closed, dormant, or proposed. Open practices are those actively prescribing, while those closed are not, comprising practices that have merged or ceased trading. Dormant and proposed practices are in a transitional state and thus removed. Practices closing between 1 April 2016 and 31 March 2019 were identified and the constituent practice population by Lower layer Super Output Areas (LSOA) gathered. LSOAs are geographically defined groupings of 1000–1500 patients, created from the 2001 census. Each LSOA is linked to multiple practices and practices linked to multiple LSOAs. Patient movements owing to practice closure are detailed in Figure 1 and Figure 2. For every LSOA, the number of patients registered at closed practices was divided by the total patients in that LSOA, providing the percentage of the LSOA exposed to closure. For each surviving practice, this percentage was then multiplied by the number of the surviving practice’s patients from that LSOA. The sum of these values for the practice’s constituent LSOAs was calculated and divided by the total practice list size to provide the closure exposure coefficient, detailed in Figure 3. The closure exposure coefficient is between 0 and 1, whereby 0 represents no exposure to closure, while 1 indicates all the practice’s patients were at a practice that closed. A closure exposure coefficient of 0.05 indicates an estimated 5% of a practice’s patients were from a closed practice.

Only English practices were included as the outcome variables are not standardised across the UK. Each practice was given a unique code to link datasets.

### Neighbourhood and demographic (confounder) data

The authors were interested in controlling for key confounders from the composition of the community. The Index of Multiple Deprivation (IMD) income deprivation score, a surrogate for deprivation using data from 2019, was gathered for each practice with the higher a score the more deprived the practice population. Rurality was defined through the binary urban or rural classification from 2011, which was based on the practice postcode.

Seventy-one of the practices had missing rurality data, so authors manually inputted this data via the postcode location. Extremes of age distribution, defined as percentage of practice population aged <4 years or aged >75 years were calculated from the March 2020 General Practice Workforce data, held by NHS Digital. Public Health England general practice ethnic group estimates were linked, which were created from a combination of 2011 census and 2015–2016 LSOA data. Collinearity was checked for confounding data using Pearson correlation coefficient.

### Funding data

Total NHS payments to general practice and unweighted list sizes was obtained from NHS England’s NHS Payments to general practice 2019–2020 dataset. This included all payments from the core contract, QOF, enhanced services, and other payment schemes.

### Workforce data

General Practice Workforce data from March 2020, held by NHS Digital, was obtained. Full-time equivalent (FTE) GP, nurse, direct patient care (DPC), and administration categories were used, as a measure of time worked. The number of patients per FTE staff was calculated.

### Quality and Outcomes Framework

The overall QOF score was gathered from NHS Digital. This is a pay-for-performance mechanism, scores of which can be used as a proxy for quality of care. Total number of QOF points was used.

### GP Patient Survey

This is annual data gathered from a sample of practice patients, which includes patient satisfaction with their registered practice. Responses are weighted to account for varying response rates across population groups. Survey data from January 2020 on patient satisfaction of phone and website access (% easy [total]), confidence in the healthcare professional (% yes [total]), and overall satisfaction (% good [total]) was used.

### Participants

The aforementioned data sources were linked with practices. Given concerns practices may maintain an active prescription code, without being an active practice, atypical practices were removed, defined as <500 (*n* = 8) or >5000 patients (*n* = 213) per FTE GP, or <750 total registered patients (*n* = 26). Sixty-three practices had <1 FTE administrative staff, including 45 practices that had none. Practices are mandated to be open from 8.30 am to 6.30 pm, meaning that these data were implausible and as such these practices were removed before analysis. This left a total of 6192 practices included.

### Statistical analysis

All data analyses were conducted in R Studio (version 1.4.1717). Collinearity was assessed using variance inflation factors. Full data availability could be seen for 92.03% of practices, with workforce variables being the most commonly missing variable, with 4.37% and 2.22% of FTE DPC and nursing data being missing, respectively. As this is <5% for each individual variable with >90% full data availability, missing data were ignored.

Mean, 95% confidence interval (CI) of the mean, and standard deviation were calculated for each covariate, detailed in Table 1. Outcome variables included unweighted list size, total practice funding, total practice funding per patient, FTE GP, unweighted list size per FTE GP, FTE nurse, unweighted list size per FTE nurse, FTE administration, unweighted list size per FTE administration, FTE DPC, unweighted list size per FTE DPC, patient satisfaction with phone access (percentage easy [total]), patient satisfaction with website access (percentage easy [total]), patient confidence with healthcare professional (percentage yes [total]), overall satisfaction with practice (percentage good [total]) and total QOF points. Meanwhile, confounding variables were percentage aged <4 years, income deprivation score, non-White ethnic group (proportion of the population not self-defining as white), and practice rurality. Univariable linear regression (logistic regression for rurality) of the closure exposure coefficient on the confounding variables was performed to analyse their confounding influence, as presented in Table 2.

**Table 1.** Characteristics of included general practices

	**Mean**	**Standard deviation**	**95% confidence interval of the mean**
Population aged <4 years (%)	5.33	1.41	5.29 to 5.36
Population >75 years (%)	8.14	3.57	8.05 to 8.23
Non-White ethnic group (%)	16.55	19.74	16.05 to 17.05
Income deprivation score	0.14	0.07	0.14 to 0.14
Total practice funding (£)	1 411 739	921 786.5	1 388 775 to 1 434 703
Unweighted population	9029.9	5684.35	8888.3 to 9171.5
Total funding per unweighted patient	159.79	51.15	158.52 to 161.07
FTE GP	5.2	3.67	5.1 to 5.3
Unweighted population per FTE GP	2013.7	811.61	1993.4 to 2033.9
FTE nurse	2.6	2.36	2.5 to 2.6
Unweighted population per FTE nurse	5112.4	4199.08	5006.4 to 5218.4
FTE administration	10.4	7.86	10.2 to10.6
Unweighted population per FTE administration	968.3	616.55	952.9 to 983.7
FTE DPC	2.0	2.49	2.0 to 2.1
Unweighted population per FTE DPC	7793.2	8507.39	7562.2 to 8024.2
Patient satisfaction with phone access (% easy [total])	69.71	20.10	69.21 to 70.21
Patient satisfaction with website access (% easy [total])	77.20	12.02	76.90 to 77.50
Overall satisfaction with practice (% good [total])	82.88	10.00	82.63 to 83.13
Confidence and trust in the healthcare practitioners (% yes [total])	95.23	37.68	95.15 to 95.33
Quality and Outcomes Framework total points (maximum 559)	534.99	31.09	534.21 to 535.76

*FTE = full-time equivalent; DPC = direct patient care.*

**Table 2.** Univariable regression results for the included confounding factors

**Variable**	**Linear regression — 10% increase in practice population from closed practice**		

	**β coefficient**	**95% confidence interval**	***P*-value**
Aged <4 years (%)	0.12	0.05 to 0.18	<0.05

Aged >75 years (%)	−0.48	−0.64 to −0.32	<0.05

Income deprivation score	0.01	0.01 to 0.01	<0.05

Non-White ethnic group	1.64	0.74 to 2.54	<0.05

**Logistic regression – rural: urban classification on closure exposure coefficient**			
Urban classification	0.07	0.02 to 0.12	<0.05

The impact of the closure exposure coefficient on the outcome variables was analysed via multiple regressions while controlling for the confounding characteristics. Each individual outcome variable was analysed in a separate multiple regression with β coefficient, 95% CIs, *P*-value, and variance inflation factor presented in Table 3.

**Table 3.** Multiple linear regression results showing a 10% increase in practice exposure to closure

**Variable**	**Multiple regression^a^ — 10% increase in practice population from closed practice**			
	**β coefficient**	**95% confidence interval**	***P*-value**	**Max VIF**
Unweighted list size	1925.6	1675.8 to 2175.4	<0.05	1.43
Total practice funding (£)	282 268.66	241 147.72 to 323 119.60	<0.05	1.43
Total practice funding per patient (£)	−2.37	−4.22 to −0.51	<0.05	1.43
FTE GP	0.7	0.6 to 0.9	<0.05	1.43
Unweighted list size per FTE GP	86.9	50.5 to 123.3	<0.05	1.43
FTE nurse	0.7	0.6 to 0.8	<0.05	1.43
Unweighted list size per FTE nurse	−61.5	−242.0 to 119.0	0.50	1.43
FTE administration	2.7	2.3 to 3.0	<0.05	1.43
Unweighted list size per FTE administration	6.4	−21.3 to 34.0	0.65	1.43
FTE DPC	0.6	0.5 to 0.7	<0.05	1.44
Unweighted list size per FTE DPC	−293.5	−697.5 to 110.6	0.16	1.47
Patient satisfaction with phone access (% easy [total])	−1.87	−2.75 to −0.99	<0.05	1.43
Patient satisfaction with website access (% easy [total])	−1.05	−1.57 to −0.53	<0.05	1.43
Confidence (% yes [total])	−0.06	−0.09 to −0.02	<0.05	1.42
Overall satisfaction with practice (% good [total])	−1.14	−1.56 to −0.72	<0.05	1.43
Quality and Outcomes Framework total points	−0.17	−1.50 to 1.16	0.80	1.43

a *Multiple regression included income deprivation score, percentage aged <4 years, percentage non-White, and rurality variables as confounders. FTE = full-time equivalent; DPC = direct patient care; VIF = variance inflation factor.*

## RESULTS

A total of 694 (8.41%) practices closed. A final sample of 6192 surviving practices were included. The average closure exposure coefficient was 0.03, ranging from 0–0.78. A total of 5137 (82.96%) were urban practices, while 1055 (17.04%) were rural. Table 1 details the characteristics of the included practices.

Table 2 details the interaction between the closure exposure coefficient and the confounding variables. Table 3 details the results of the multiple linear regression. The effect of a 0.1 increase in the closure exposure coefficient is presented, representing a 10% increase in practice population exposed to closure. Practices with increased exposure to closure had a significantly greater proportion of their patients aged <4 years, increased income deprivation, non-White ethnicity, and in urban settings, while fewer of their patients were aged >75 years. Strong negative correlation was observed between being aged >75 years and non-White ethnicity (−0.63) and aged <4 years (−0.54) and thus was not included in the regression equations.

### Population size and funding

A 0.1 increase in closure exposure coefficient resulted in 1925.6 (95% CI = 1675.8 to 2175.4) more patients per practice and a disproportionate £282 268.66 (95% CI = £241 147.72 to £323 119.60), increase in funding, with a £2.37 (95% CI = £4.22 to £0.51) reduction in funding per patient (Table 3).

### Workforce

A 0.1 increase in closure exposure coefficient resulted in an increase in all FTE staff types (FTE GP = 0.7 [95% CI = 0.6 to 0.9], FTE nurse = 0.7 [95% CI = 0.6 to 0.8], FTE administration = 2.7 [95% CI = 2.3 to 3.0], and FTE DPC = 0.6 [95% CI = 0.5 to 0.7]). However, when taking account of patient increases, this showed a mixed picture, with significantly more patients per FTE GP 86.9 (95% CI = 50.5 to 123.3) while no significant change in patients per other staff type was found (Table 3).

### Quality (univariate then multivariate)

A 0.1 increase in closure exposure coefficient resulted in significantly lower patient satisfaction scores throughout all variables (phone: −1.87 [95% CI = −2.75 to −0.99], website: −1.05 [95% CI = −1.57 to −0.53], confidence: −0.06 [95% CI = −0.09 to −0.02], overall: −1.14 [95% CI = −1.56 to −0.72]). No significant change in QOF score was found (Table 3).

## DISCUSSION

### Summary

Practice closures are associated with a decrease in funding per patient and reduced patient satisfaction within surviving practices. Increases in patient list size resulted in an increase in the number of patients per FTE GP.

Patient and funding changes may be owing to consolidation of patients in the surviving practices. The £2.37 (1.48%) reduction in funding per patient with increasing exposure to closure suggests inequality may be exacerbated by closures, although this change was small. The changes in the number of patients per GP may be the result of GPs from closing practices reducing or stopping practising in the geographic area. The universal reduction in satisfaction may indicate declines in the quality of the service. However, QOF remained stable so no change in clinical quality has been observed.

### Strengths and limitations

The study utilised key datasets as well as age profile, ethnic group, rurality, and deprivation, which are important confounding variables. This enabled the analysis of the association between practice funding, workforce, and quality with practice closures.

As with all cross-sectional studies, reverse causation needs to be considered. Areas with lower patient satisfaction, proportionate funding, or proportionate workforce may be predisposed to closure.

Patient need and associated resource requirements vary between practices. This is addressed in UK primary care by weighting the list size using the Carr-Hill formula.^20^ Unweighted patient list size was chosen owing to the probable correlation with confounders that the weighting mechanism uses. Further, the weighting mechanism is commonly criticised for failing to address differences in need.^21^

GP Patient Survey data are commonly used in this field of research. This is because the data are public availability and are considered reliable.^22^ However, it is important to be cognisant of the influence of the low response rate.^16^ Total QOF score is an aggregate of different domains. Further insights may be gained by analysing the constituent QOF domains.

### Comparison with existing literature

No studies have been conducted on the consequences of practice closure in English general practice. Different primary care physicians have different practising styles, which results in changed utilisation patterns when patients move.^14^^,^^15^^,^^17^^,^^18^ Utilisation is not directly measured here, but this may be reflected in the reduced satisfaction with remaining services.

Research on continuity of care is more advanced. Disruption in continuity can be expected from practice closures. Improvements in patient satisfaction and outcomes are observed with improved continuity of care.^23^ This may partly explain the reduced satisfaction observed with practice closures.

### Implications for research and practice

While pressure in UK general practice is recognised,^19^ closures of practices is under-researched. This study identifies that practice closures change practice funding, workforce, and quality.

Surviving practices are at risk of relative decline in funding, GP supply, and patient satisfaction. Therefore, closures may make providing care in these practices more challenging, increasing the risk of supply— demand imbalance and associated access issues.^24^ Alternatively, given QOF remains stable, findings may indicate efficiency improvements; but declines in satisfaction contradicts this indication. Given closures are more common among practices serving deprived and non-White populations, they may widen existing inequalities.^21^^,^^25^ Practices and commissioners may be able to mitigate for the consequences of closures through policy changes, such as preventing the closure, or increasing resources, for surviving practices. However, there is currently minimal evidence on how best to intervene.

Practice closure describes a broad set of organisational changes. These findings may alter by closure definition; therefore, it is important to study the individual closure mechanisms for their associated consequences. Understanding the causes of the different closure definitions would also be helpful in healthcare planning.

Similarly, this study used broad workforce and quality indicators. If workforce outcomes are disaggregated, such as by nursing type, this would develop a deeper understanding of aspects such as substitution. Similarly, these results may not translate into deleterious patient outcomes, which needs to be studied.

## Data

Data gathered from publicly available sources as detailed in the manuscript.

## References

[1] Mohamoud A Almost 800 GP practices have shut over the past eight years.

[2] Headd B (2003). Redefining business success: distinguishing between closure and failure. Small Business Economics.

[3] Roberts A, Wallace W, Moles P (2016). Mergers and Acquisitions.

[4] Bazzoli GJ, Andes S (1995). Consequences of hospital financial distress. Hosp Health Serv Adm.

[5] British Medical Association (2022). GP contract changes England 2022/23.

[6] Khan N, Rudoler D, McDiarmid M, Peckham S (2020). A pay for performance scheme in primary care: meta-synthesis of qualitative studies on the provider experiences of the quality and outcomes framework in the UK. BMC Fam Pract.

[7] Checkland K, McDermott I, Coleman A (2018). Planning and managing primary care services: lessons from the NHS in England. Public Money & Management.

[8] Bostock N Average GP practice list tops 9,000 after 30% rise in seven years.

[9] Forbes JL, Forbes H, Sutton M (2020). Changes in patient experience associated with growth and collaboration in general practice: observations study using data from the UK GP Patient Survey. Br J Gen Pract.

[10] Morciano M, Checkland K, Hammond J (2020). Variability in size and characteristics of primary care networks in England: observational study. Br J Gen Pract.

[11] Iacobucci G (2017). Rights and responsibilities when a general practice closes. BMJ.

[12] Gravelle H, Liu D, Santos R (2022). How do clinical quality and patient satisfaction vary with provider size in primary care? Evidence from English general practice panel data. Soc Sci Med.

[13] Bischof T, Kaiser B (2021). Who cares when you close down? The effects of primary care practice closures on patients. Health Econ.

[14] Simonsen M, Skipper L, Skipper N, Thingholm PR (2021). Discontinuity in care: practice closures among primary care providers and patient health care utilization. J Health Econ.

[15] Fadlon I, Van Parys J (2020). Primary care physician practice styles and patient care: evidence from physician exits in Medicare. J Health Econ.

[16] Piwnica-Worms K, Staiger B, Ross JS (2021). Effects of forced disruption in Medicaid managed care on children with asthma. Health Serv Res.

[17] Kwok J (2019). How do primary care physicians influence healthcare? Evidence on practice styles and switching costs from Medicare. SSRN.

[18] Shwab S You had me at hello: the effects of disruptions to the patient—physician relationship.

[19] Baird B, Charles A, Honeyman M Understanding pressures in general practice.

[20] British Medical Association (2020). Global sum allocation formula.

[21] Ashworth M, L’Esperance V, Round T (2021). Primary care funding entrenches health inequalities: time for a rethink. Br J Gen Pract.

[22] Lyratzopoulos G, Elliot M, Barbiere J (2011). How can health care organisation be reliably compared? Lessons from a national survey of patient experience. Med Care.

[23] Jeffers H, Baker M (2016). Continuity of care: still important in modern-day general practice. Br J Gen Pract.

[24] Abel GA, Gomez-Cano M, Mustafee N (2020). Workforce predictive risk modelling: development of a model to identify general practices at risk of a supply-demand imbalance. BMJ.

[25] Watt G (2018). The inverse care law revisited: a continuing blot on the record of the National Health Service. Br J Gen Pract.

